# Supplementary figures and images for: Revealing non-trivial information structures in aneural biological tissues via functional connectivity
Source: PLoS Comput Biol. 2025 Apr 14;21(4):e1012149. doi: 10.1371/journal.pcbi.1012149 (PMC11996219; doi:10.1371/journal.pcbi.1012149)

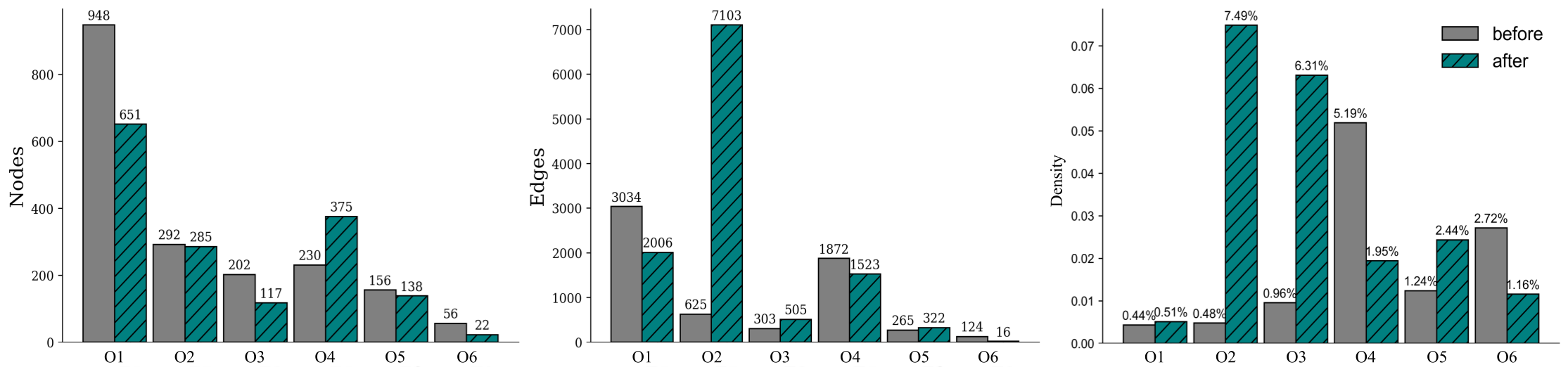

Supplement: S1 Fig — Number of Nodes, Edges, and Network Density for each video. (TIFF) [file pcbi.1012149.s001.tiff]
